# Trends in the clinical characteristics of HIV-infected patients initiating antiretroviral therapy in Kenya, Uganda and Tanzania between 2002 and 2009

**DOI:** 10.1186/1758-2652-14-46

**Published:** 2011-09-28

**Authors:** Elvin H Geng, Peter W Hunt, Lameck O Diero, Sylvester Kimaiyo, Geofrey R Somi, Pius Okong, David R Bangsberg, Mwebesa B Bwana, Craig R Cohen, Juliana A Otieno, Deo Wabwire, Batya Elul, Denis Nash, Philippa J Easterbrook, Paula Braitstein, Beverly S Musick, Jeffrey N Martin, Constantin T Yiannoutsos, Kara Wools-Kaloustian

**Affiliations:** 1 Department of Medicine, San Francisco General Hospital, University of California at San Francisco, 995 Potrero Avenue, San Francisco, USA; 2 Department of Medicine, Indiana University, 410 West 10th Street, Indianapolis, USA; 3 Department of Medicine, Mbarara University of Science and Technology, 1410 University Road, Mbarara, Republic of Uganda; 4 National AIDS Control Program, Dar es Salaam, P.O.Box 11857, The United Republic of Tanzania; 5 Infectious Diseases Institute, P.O. Box 22418, Kampala, Republic of Uganda; 6 Department of Medicine, Moi University, Eldoret, Kenya; 7 Massachusetts General Hospital Center for Global Health, Harvard Medical School, 641 Huntington Avenue, Boston, MA, USA; 8 St. Francis Hospital, Nsambya Hill, Box 7146, Kampala, Republic of Uganda; 9 Makerere University-Johns Hopkins University Research Collaboration, Republic of Uganda; 10 Kisumu MTCT-Plus Initiative, Kisumu, Republic of Kenya; 11 International Center for AIDS Care and Treatment Programs, 722 W168th Street, New York, NY, USA

## Abstract

**Background:**

East Africa has experienced a rapid expansion in access to antiretroviral therapy (ART) for HIV-infected patients. Regionally representative socio-demographic, laboratory and clinical characteristics of patients accessing ART over time and across sites have not been well described.

**Methods:**

We conducted a cross-sectional analysis of characteristics of HIV-infected adults initiating ART between 2002 and 2009 in Kenya, Uganda and Tanzania and in the International Epidemiologic Databases to Evaluate AIDS Consortium. Characteristics associated with advanced disease (defined as either a CD4 cell count level of less than 50 cells/mm^3 ^or a WHO Stage 4 condition) at the time of ART initiation and use of stavudine (D4T) or nevirapine (NVP) were identified using a log-link Poisson model with robust standard errors.

**Results:**

Among 48, 658 patients (69% from Kenya, 22% from Uganda and 9% from Tanzania) accessing ART at 30 clinic sites, the median age at the time of ART initiation was 37 years (IQR: 31-43) and 65% were women. Pre-therapy CD4 counts rose from 87 cells/mm^3 ^(IQR: 26-161) in 2002-03 to 154 cells/mm^3 ^(IQR: 71-233) in 2008-09 (p < 0.001). Accessing ART at advanced disease peaked at 35% in 2005-06 and fell to 27% in 2008-09. D4T use in the initial regimen fell from a peak of 88% in 2004-05 to 59% in 2008-09, and a greater extent of decline was observed in Uganda than in Kenya and Tanzania. Self-pay for ART peaked at 18% in 2003, but fell to less than 1% by 2005. In multivariable analyses, accessing ART at advanced immunosuppression was associated with male sex, women without a history of treatment for prevention of mother to child transmission (both as compared with women with such a history) and younger age after adjusting for year of ART initiation and country of residence. Receipt of D4T in the initial regimen was associated with female sex, earlier year of ART initiation, higher WHO stage, and lower CD4 levels at ART initiation and the absence of co-prevalent tuberculosis.

**Conclusions:**

Public health ART services in east Africa have improved over time, but the fraction of patients accessing ART with advanced immunosuppression is still high, men consistently access ART with more advanced disease, and D4T continues to be common in most settings. Strategies to facilitate access to ART, overcome barriers among men and reduce D4T use are needed.

## Background

An unprecedented global effort to provide antiretroviral therapy (ART) to HIV-infected patients in resource-limited settings is underway. Led by the Global Fund to Fight AIDS, Tuberculosis and Malaria, established in 2003, and the US President's Emergency Fund for AIDS Relief (PEPFAR), founded in 2004, US$50 billion had been invested in global HIV/AIDS care, treatment and prevention by 2009 [1]. As a result, 5 million HIV-infected persons in resource-limited settings have started ART, and the World Health Organization (WHO) estimates that in sub-Saharan Africa, 1.2 million lives and 2.3 million life-years have been saved [2].

In east Africa, ART coverage based on initiation using previous WHO criteria of CD4 counts of less than 200 cells/mm^3 ^or 200-350 cells/mm^3 ^with select conditions has risen from less than 5% in 2002 to 65% in Kenya, 53% in Uganda and 44% in Tanzania by 2009 [3]. Access to ART has also had a measureable impact on the economic and social dimensions of life in Africa by increasing labour capacity [4], maintaining educators [5], increasing survival of children [6] and even raising educational attainment among children in households affected by HIV [7]. Finally, the scale up has proven that large-scale access to complex, potentially toxic, life-long ART can be achieved in resource-limited settings - a task that some experts considered implausible not long ago [8].

Despite indisputable evidence of successes, more information about the characteristics of patients starting ART in "real-world settings" - and how those characteristics are changing over time - is needed to characterize "gaps" in access to ART in east Africa. Trends in the CD4 levels and WHO stage at the time of ART initiation can reveal the extent to which care is reaching patients before advanced disease and the attendant high risk of early mortality due to concurrent opportunistic infections [9,10]. Demographic characterization of patients starting ART over time may yield information about socio-behavioural groups who face barriers to care [11].

From a health systems perspective, examining the changing characteristics of care, such as the fraction of patients who self-pay for ART and the travel time from home to clinic, over time can provide an understanding of structural obstacles to care [12,13]. Finally, characterizing trends in access to ART services must also include a consideration of the specific medications that are being used. Nevirapine (NVP) was chosen as the non-nucleoside reverse transcriptase inhibitor (NNRTI) of choice despite higher toxicity than efavirenz because of substantially lower costs. The global roll out initially relied heavily on stavudine (D4T), a relatively toxic drug which is being phased out; monitoring changes in the use of D4T in first-line regimens over time is needed to demonstrate progress [14].

To date, however, no reports contain enough data on enough patients over enough time to provide a regionally representative picture of patients starting ART in east Africa. The East Africa International Epidemiologic Databases to Evaluate AIDS (EA-IeDEA) is a consortium of clinic-based cohorts in Kenya, Uganda and Tanzania that captures data from "real-world" settings in diverse environments. Using data from 2002-2009, we describe the epidemiologic characteristics of patients accessing ART during this phase of the scale up in east Africa.

## Methods

### Patients

The EA-IeDEA is a cohort of patients from clinical care sites in Uganda, Kenya and Tanzania. IeDEA seeks to harmonize data from HIV care and treatment sites in order to evaluate the effectiveness of the ART roll out using data collected in representative "real-world settings" (i.e., in high-volume clinics providing care without access to routine HIV RNA monitoring, staffed and stocked by national ministries of health, or where an implementing partner is implementing the "public health approach"[15]).

In Kenya, participating programmes include the United States Agency for International Development-Academic Model Providing Access to Healthcare (USAID-AMPATH), the Family AIDS Care and Education Services based in Nyanza Province and Nyanza Provincial Hospital. In Uganda, affiliated sites include the Infectious Diseases Institute and Mulago Hospital, the Nsambya Hospital in Kampala and the Immune Suppression Syndrome Clinic in Mbarara, which is located in the rural southwest part of the country. Contributing sites in Tanzania include the Tumbi Regional Hospital in Kibaha, the Ocean Road Cancer Institute in Dar es Salaam and the Morogoro Regional Hospital in Morogoro.

We evaluated data from all adult, treatment-naïve patients starting ART between 2002 and 2009; patients who were exposed to ART for prevention of mother to child transmission (PMTCT) were included. The exact date of database closure differed by clinic site and ranged from 31 March 2008 to 19 May 2009.

### Measurements

Socio-demographic, clinical, medication and laboratory data were collected in the course of routine clinical care by providers on standardized forms specific to each of the sites. Information collected on paper charts is manually entered into electronic databases by data entry clerks. Prospective data quality control mechanisms to optimize accuracy and reduce missing data are a part of data collection at all IeDEA sites and all employ one or more of the following procedures: data entry range restrictions; sampling of charts to identify missing and erroneous data; and reconciliation of errors and missing information with clinicians and primary records. At every site, period audits are conducted by the regional data centre.

We defined the pre-therapy CD4 value as the last CD4 determination within six months of ART initiation. The pre-therapy WHO stage (which was routinely determined at all sites) was defined as the maximum WHO stage documented for the patient before ART initiation. "Programme" is defined by a single administrative unit and "site" is defined by a single physical location for ART services. For example, a programme (e.g., USAID-AMPATH) may have multiple sites (e.g., Eldoret, Burnt Forest and Kitale). Tuberculosis (TB) was considered present if either an active TB diagnosis was present at ART initiation or if the patient was receiving anti-TB therapy at the time of ART initiation. Continuous variables were discretized according to convention (CD4 count cut offs were made at 50, 100 and 200 cells/mm^3^) or were split into quartiles (as in the case of age). The joint effect of sex and history of PMTCT was handled as a nominal categorical variable with levels equal to: (1) women with no history of PMTCT; (2) men; and (3) women with a history of PMTCT.

### Analysis

We conducted cross-sectional analyses of socio-demographic, clinical and laboratory characteristics of patients at the time they accessed ART, stratified by calendar year of ART initiation, sex and across country to evaluate temporal and regional trends. Because of the large number of comparisons (for example, of differences in a given patient characteristic across strata of time and country) we used graphical representation of the data whenever possible. Statistical comparisons of continuous variables across categorical groups were conducted with analyses of variance (ANOVA) or linear regression. We conducted single predictor and multivariable analyses to identify factors associated with: (1) advanced immune-suppression (defined as a patient with either a CD4 level of less than 50 cells/mm^3 ^or WHO Stage 4 condition) at the time of ART initiation; (2) receipt of D4T in the first-line regimen; or (3) receipt of nevirapine (NVP) in the first-line regimen.

In the subset of patients where data were available, we examined factors associated with self-pay using logistic regression. Predictor selection for multivariable models was driven by substantive knowledge, as well as the desire to include both individual-level predictors (e.g., age and sex) and ecological predictors (e.g., year of ART initiation, country and programme) to create "multi-level" models and to reduce confounding [16,17]. To obtain more interpretable risk ratios, we used a log-link Poisson model, with robust standard errors to avoid the resulting misspecification of the model standard errors and to account for clustering by site [18]. All analyses were conducted in Stata version 11 (Stata Corporation, College Station, TX). This study was reviewed and approved by institutional review bodies of all participating sites and universities.

## Results

**Figure 1:**
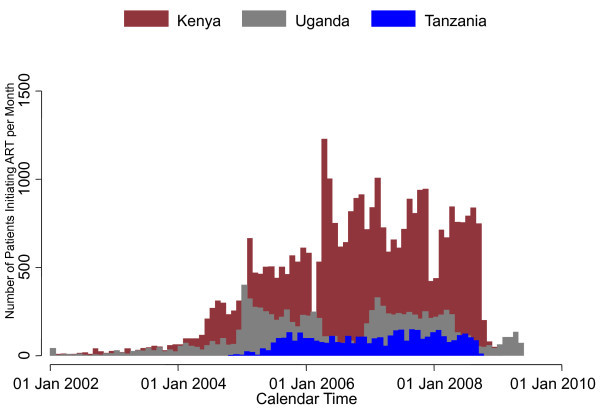
**The number of patients starting ART in the East Africa IeDEA Consortium each month, stratified by country**.

In total, 48, 658 adult patients from 35 clinic sites and from 10 programmes were included in this analysis. The median number of patients at each clinic site was 788 (IQR: 342 to 1816) and in each programme was 1283 (range: 292-32, 221). Of the total patients, 33, 680 (69%) were from Kenya, 10, 859 (22%) were from Uganda, and 4119 (9%) were from Tanzania. Of 35 sites, 28 were in Kenya, four in Uganda and three in Tanzania. Overall, 1118 (2%) patients started ART in 2002-03, 12, 875 (27%) in 2004-05, 24, 811 (51%) in 2006-07, and 9854 (20%) in 2008-09 (Figure 1).

### Socio-demographic characteristics

The median age at the time of ART initiation was 37 years (IQR: 31-43). When stratified by country, patients in Uganda were slightly younger overall with a median age of 36 years (IQR: 31-41) compared with those in Kenya (median 37 years, IQR: 31-44) and in Tanzania (median 38 years, IQR: 32-45). Overall, men who started ART were older than women, with the average difference most pronounced in Tanzania (5.0 years, 95% CI: 4.4-5.7) compared with Uganda (4.2 years, 95% CI: 3.8-4.5) or Kenya (4.2 years, 95% CI: 4.0-4.4). The majority of patients (65%) were women. Over time, men comprised a decreasing fraction of new patients accessing ART in Kenya, dropping from 41% in 2002-03 to 34% in 2008-09 (p < 0.01), but the fraction of men accessing care in Uganda and Tanzania did not change markedly.

### Clinical characteristics

**Figure 2:**
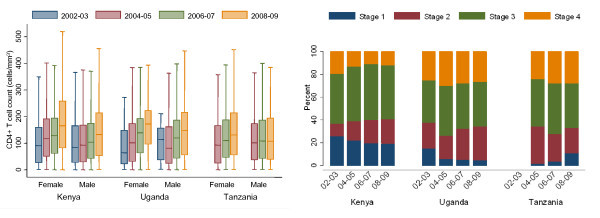
**Level of immunosuppression as measured by CD4 level and WHO stage at the time of ART initiation in the East Africa IeDEA Consortium, stratified by time, country and (for CD4 levels) sex**. Pre-therapy CD4 value is missing in 7283/48, 658 (15%). WHO stage is missing in 2648/48, 658 (5%).

Overall, across time, 85% of patients had a pre-therapy "baseline" CD4 determination, and the median was 122 cells/mm^3 ^(IQR: 52 to 193). When stratified by country, calendar time and sex, several trends are apparent. First, the median pre-therapy CD4 counts rose over time from 87 cells/mm^3 ^(IQR: 26-161) in 2002-03 to 105 cells/mm^3 ^(IQR: 38-179) in 2004-05, 121 cells/mm^3 ^(IQR: 54- 189) in 2006-07 and 154 cells/mm^3 ^(IQR: 71-233) in 2008-09 (p < 0.001). Second, the pre-therapy CD4 counts summarized over all time points were on average higher in women at 130 cells/mm^3 ^(IQR: 59-198) than in men at 107 cells/mm^3 ^(IQR: 40-181) (p < 0.001). Third, the rise is most marked among women in Kenya and least among men in Tanzania (Figure 2).

Overall, 95% of patients had pre-therapy WHO stage documented: of those, 16% were Stage 1, 21% were Stage 2, 46% were Stage 3 and 17% were Stage 4. The fraction of patients with WHO Stage 4 disease at the time of accessing ART was lower in Kenya (12%) than in Uganda (28%) and Tanzania (27%) (p < 0.001). The percentage of patients with WHO Stage 4 conditions declined significantly in Kenya from 20% in 2002-03 to 12% in 2008-09 (p for trend < 0.001), but rose in Uganda from 33% to 36% (p for trend = 0.02) in the same period. No change was seen in Tanzania (p for trend = 0.21) (Figure 2). Presentation with advanced disease (i.e., a CD4 count < 50 cells/mm^3 ^or WHO Stage 4) in 2002-03 was 26%, 35% in 2004-05, 31% in 2006-07 and 27% in 2008-09.

Multivariable analyses found male sex (compared with women with no history of PMTCT exposure), calendar year, residence in Uganda and Tanzania and self-pay were associated with accessing ART at advanced disease. In contrast, women with a history of treatment for PMTCT (compared with women with no history of treatment for PMTCT) and older age were associated with decreased risk of advanced disease at the time of accessing ART (Table 1).

**Table 1:** Characteristics associated with advanced disease (defined as either a CD4 level ≤ 50 cells/mm3 or a WHO Stage 4 condition) at the time of ART initiation using a log-link Poisson model with robust standard errors.

Factor	Number (%) (n = 48, 658)	Prevalence ratio	95% confidence interval	P value
Sex*				
Women without a history of PMTCT	29, 939	Ref.		
Men	17, 173	1.33	1.26-1.41	< 0.001
Women with a history of PMTCT	1440	0.59	0.36-0.95	0.03

Calendar year of ART initiation, (per 2 years)		0.92	0.86-0.99	0.03

Country of residence				
Kenya	33, 680	Ref.		
Uganda	10, 859	1.28	1.04-1.57	0.04
Tanzania	4119	1.38	1.00-1.92	0.05

Age at ART initiation (years)				
Quartile 1 (18-31)	13, 152	Ref.		
Quartile 2 (32-37)	12, 766	0.98	0.82-1.04	0.44
Quartile 3 (38-43)	10, 662	0.97	0.89-1.05	0.40
Quartile 4 (44-88)	12, 078	0.85	0.79-0.91	< 0.001

Self-pay+				
No	46, 479	Ref.		
Yes	574	1.41	1.24-1.59	< 0.001

Each factor is adjusted for all other factors in the table (N = 48, 658)

* Missing in 106 of 48, 658 (0.2%)

+ Missing in 1605 (3.3%)

### ART medication use

Among initial regimens which were available in 96% of patients, stavudine (D4T) was the most common nucleoside reverse-transcriptase inhibitor component during the period of study: overall, 76% of patients started ART regimens that contained D4T. The proportion of patients starting regimens with D4T actually rose between 2002-03 (78%) and 2004-05 (88%), but then fell in 2006-2007 (76%) and in 2008-2009 (59%).

**Figure 3:**
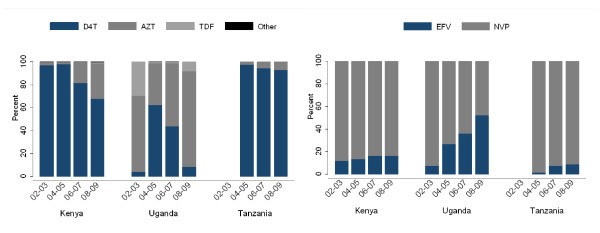
**Composition of non-lamivudine component of nucleoside reverse transcriptase inhibitor and non-nucleoside reverse transcriptase inhibitor in the initial ART regimen among HIV-infected patients in the East Africa IeDEA Consortium, stratified by time and country**. First regimen is missing in 1879/48, 658 (4%).

In Kenya, D4T use fell consistently from 97% in 2002-03 to 68% in 2008-09 (p for trend < 0.001). In Uganda, the fraction of initial regimens containing D4T rose between 2002-03 and 2004-05 from 6% to 63%, but subsequently fell to 44% in 2006-07 and to 8% in 2008-09 (p for trend < 0.001). Of note, in Uganda during the self-pay era, tenofovir was common, which disappeared during the early days of the free ART era and more recently has re-emerged: by 2008-09, 8% of patients starting ART used tenofovir-based regimens. In Tanzania, the reduction of D4T use has been less apparent: in 2004-05, 98% of initial regimens contained D4T and in 2008-09, the fraction fell to 93% (p for trend < 0.001) (Figure 3a). In a multivariable model, higher WHO stage was associated with a higher chance of D4T use in the first regimen, whereas male sex, later calendar time, residence in Uganda, higher CD4 cell count and TB were associated with reduced use of D4T (Table 2).

**Table 2:** Factors associated with the use of D4T in the first ART regimen in multivariable analysis using log-link Poisson model with robust standard errors.

Factor	Number (%) (n = 48, 658)	Risk ratio	95% Confidence interval	P value
Male sex*	17, 173	0.97	0.95-0.99	< 0.001

Year of ART initiation (per 2 years)		0.81	0.73-0.89	< 0.001

Country of residence				
Kenya	33, 680	ref		
Uganda	10, 859	0.49	0.33-0.72	< 0.001
Tanzania	4119	1.14	1.09-1.21	< 0.001

Age at ART initiation (years)				
Quartile 1 (18-31)	13, 152	ref		
Quartile 2 (32-37)	12, 766	1.00	0.99-1.02	0.58
Quartile 3 (38-43)	10, 662	1.00	0.99-1.03	0.52
Quartile 4 (44-88)	12, 078	0.99	0.97-1.02	0.56

WHO stage^+^				
Stage 1	7263	ref		
Stage 2	9808	1.01	0.98-1.05	0.52
Stage 3	21, 291	1.06	1.02-1.11	< 0.01
Stage 4	7648	1.10	1.02-1.07	0.01

Pre-therapy CD4 level^ ± ^				
≤ 50 cells/mm^3^	10, 148	ref		
51-100 cells/mm^3^	7377	0.97	0.89-1.06	0.27
101-200 cells/mm^3^	14, 528	0.87	0.81-0.94	0.09
> 200 cells/mm^3^	16, 605	0.86	0.80-0.93	0.02

Presence of tuberculosis		0.94	0.90-0.98	< 0.01

Each factor is adjusted for all other factors in the table.

* Missing in 106 (0.2%)

+ Missing in 2648 (5.4%)

± Missing in 7283 (15.0%)

For the NNRTI component of the initial regimens, overall NVP was used in 80% of the regimens. The proportion of NVP-containing regimens fell from 83% in 2002-03 to 74% in 2008-09 (p for trend < 0.001). In Kenya, the decrease in NVP use fell from 91% in 2002-03 to 81% in 2008-09. In Uganda, NVP use rose from 53% in 2002-03 to 72% in 2004-05 as free ART programmes scaled up, but then subsequently fell to 46% in 2008-09 (p for trend < 0.001). In Tanzania, although NVP use has decreased steadily, the overall fraction of NVP use remained high: in 2002-03, 99% used NVP and in 2008-09, the figure was 91% (Figure 3b). In multivariable analysis, year of ART initiation and pre-therapy CD4 count levels between 50 cells/mm^3 ^and 200 cells/mm^3 ^were associated with NVP use, whereas older age, male sex, higher WHO stage and TB diagnosis were associated with a reduced probability of NVP use (Table 3).

**Table 3:** Factors associated with the use of NVP in the first ART regimen using a log-link Poisson model with robust standard errors.

Factor	Risk ratio	95% Confidence interval	P value
Male sex*	0.95	0.93-0.97	< 0.01

Calendar year of ART initiation (per 2 years)	0.94	0.89-0.99	0.03

Country			
Kenya	ref		
Uganda	0.71	0.60-0.84	< 0.01
Tanzania	1.06	1.01-1.12	0.01

Age at ART initiation (years)			
Quartile 1 (18-31)	Ref.		
Quartile 2 (32-37)	0.98	0.96-1.00	0.04
Quartile 3 (38-43)	0.97	0.95-1.00	0.04
Quartile 4 (44-88)	0.98	0.96-1.01	0.14

WHO stage at ART initiation^+^			
Stage 1	Ref.		
Stage 2	1.02	0.99-1.05	0.09
Stage 3	0.98	0.96-1.01	0.46
Stage 4	0.91	0.70-0.86	< 0.01

Pre-therapy CD4 level^ ± ^			
≤ 50 cells/mm^3^	Ref.		
51-100 cells/mm^3^	1.04	1.02-1.08	< 0.01
101-200 cells/mm^3^	1.07	1.00-1.13	0.02
> 200 cells/mm^3^	1.03	0.99-1.07	0.14

Presence of tuberculosis	0.72	0.70-0.75	< 0.01

Each factor is adjusted for all other factors in the table.

* Missing in 106 (0.2%)

+ Missing in 2648 (5.4%)

± Missing in 7283 (15.0%)

### System of care

Of 46, 479 patients with known information about payment for ART, 574 (1.2%) paid for ART. The fraction of patients who paid peaked at 18% in 2003 and then quickly fell to 12% in 2004, less than 1% in 2005 and zero after 2005. Before 2006, when self-pay was completely phased out, men were more likely to pay for ART than women (OR = 1.5, 95% CI: 1.2-1.74).

Travel time from home to clinic was available for patients attending one programme in Kenya. Within this group over all time periods, 27% of patients required less than 30 minutes to travel to a clinic, 32% 31-60 minutes, 24% one to two hours, and 17% more than two hours to get to a clinic. Overall, the fraction of patients requiring more than two hours to access a clinic fell over time from 28% in 2002-03 to 15% 2008-09. This corresponds with a period when the number of clinic sites in the programme increased from 15 to 23. During the earliest time period, a smaller proportion of women required more than two hours to get to a clinic (26%) than men (30%), (p < 0.001). By 2008-09, however, the proportion was equivalent at 14%.

## Discussion

This analysis, including nearly 50, 000 patients, over eight years and covering a network of 30 sites in three countries, suggests that in addition to rapid expansion, the roll out of ART services for HIV-infected patients in east Africa is improving in effectiveness. First, we documented that the median CD4 count at the time of ART initiation in the east Africa region has increased substantially from 87 cells/mm^3 ^in 2002-03 to 185 cells/mm^3 ^in 2008-09. Second, we found improvements in the pharmaco-epidemiology of the roll out over time with reductions in more toxic regimens: the use of D4T in the first regimen fell from a peak of 88% to 58% in 2008-09, and the fraction of patients starting NVP-based regimens decreased as more regimens made use if EFV instead, even after taking into account changing trends in sex of patients starting ART over time.

Third, the fraction of patients who had longer travelling times to a clinic declined by 50% in the programme from which we had these data available, and this likely reflects the ongoing process of decentralization of ART services, a key element of improving access to ART services. Lastly, our analysis underlines that, after 2005 when the Global Fund and PEPFAR began supporting large-scale ART programmes, self-pay for ART, which has been shown to be associated with poor outcomes in Africa [19], essentially disappeared.

This analysis, which covers a large span of both time (indeed, capturing data before the rapid Global Fund- and PEPFAR-funded rise in ART availability in 2004-05) and geographic regions, also presents the opportunity to identify "gaps" in access to ART in east Africa. Existing literature has suggested that more patients with advanced disease at ART centres are men [20]. One explanation is that many asymptomatic women with high CD4 counts are detected when screened during pregnancy and referred to ART centres.

Although we also found male sex to be associated with advanced disease at presentation (defined as either WHO Stage 4 or a CD4 count of ≤ 50 cells/mm^3^), we also found that a history of PMTCT did not explain all of this association. Men were 50% more likely to access ART with advanced disease compared with women without a history of PMTCT. But women without a history of PMTCT were nearly twice as likely to access ART with advanced disease compared with women with a history of PMTCT. Because most women tested during pregnancy receive some form of PMTCT, it appears that socio-behavioural characteristics of men in east Africa confer additional risk of having advanced disease that is not completely explained by public health services targeting pregnant women. Further evaluation of the causal reasons for the association between late disease presentation and male sex among patients accessing ART is required.

Although the magnitude of the differences were not large, patients in Uganda and Tanzania in this analysis were more likely to start ART with advanced disease as compared with Kenya after adjustment for socio-demographic factors and calendar time. This is likely explained by the fact that the Tanzanian scale up of ART services occurred slightly later and coverage has only recently begun to catch up with - a history that is consistent with a programme seeing more advanced disease at ART initiation.

In Uganda, the risk of presentation with more advanced disease is driven by a larger fraction of patients with a WHO Stage 4 condition despite approximately equal CD4 cell count levels. This association may be due to differential clinical assignment of WHO stage when definitive diagnoses cannot always be made. Further study, in cohorts where definitive diagnoses are available, are needed to exclude the possibility that Ugandan patients have a higher disease burden even after accounting for CD4 count levels. Overall, when compared with recent reports from southern Africa, the demographics in our patients in east Africa are similar (i.e., proportion of women and age). The median CD4 count at ART initiation, however, is slightly higher at 122/mm^3 ^in our analysis as compared with 103/mm^3 ^in South Africa during a similar period. This likely reflects the higher burden of disease in the epidemic population in South Africa [21]. Furthermore, the CD4 count at ART initiation in our study during 2008-09 period was 154 cells/mm^3^, which is not far from contemporaneous figures of 187 cells/mm^3 ^in the United States, 159 cells/mm^3 ^in Brazil and 157 cells/mm^3 ^in China [22].

Given the increasing consensus of the high D4T toxicity, crystallized in the WHO 2010 recommendations [14], monitoring declines in D4T use is an important objective of contemporary pharmaco-epidemiology. A "gap" we observed is the marked differences in the use of D4T and NVP over time and across countries with similar economic resources. Although the fraction of patients starting D4T-based regimens declined in all countries, programmes in Uganda moved away from D4T earlier and more extensively than those in Kenya and Tanzania, and by 2009, included nearly 8% of regimens, which contained tenofovir.

The reasons for these differences, including the differences in healthcare systems and national policies, across countries with similar economic resources available for health require further explanation and research. In particular, interdisciplinary research focused on the economic, programmatic and policy issues that inform national implementation programmes may yield further explanations. Furthermore, the cost effectiveness of these differences should be quantified: although countries using less D4T may have higher per patient costs in the short run, the long-term quantification and prevention of morbidities associated with prolonged D4T use must also be assessed.

Our analysis of factors associated with specific ART drugs reflected, in general, rational drug choices and are reassuring from a public health perspective. We found several notable associations with D4T use. Male sex, older age, higher pre-therapy CD4 counts and TB were associated with a reduced risk of D4T use. Lower D4T use in men and at higher CD4 count levels may be explained by the lower prevalence of anaemia in men and in healthier patients, and hence the absence of a contraindication to zidovudine (AZT) use. Reduced D4T use in patients with TB is potentially explained by the desire to avoid the neurotoxic combination of isoniazid and D4T [23]. The observed preponderance of efavirenz (EFV) use in men likely reflects the desire to avoid EFV in women who desire children, and the elevated use of EFV in those with TB reflects recognition of the interaction between NVP and rifampin [24,25].

A limitation of this manuscript is the cross-sectional nature of the analyses. In cross-sectional studies, inferences must be made with care because time ordering is not possible and selection bias is difficult to control. For example, the finding that more men have advanced disease among patients accessing ART leads to the nominal inference that the socio-behaviour characteristics of HIV-infected men in east Africa prevents them from seeking care. However, an alternative may be considered: if the distribution of advanced disease is similar in men and women in the community (a plausible assumption especially early in the roll out when few patients were accessing ART), then the observation that male sex is associated with at advanced disease among patients accessing ART could imply that female sex is associated with advanced disease in patients unable to access ART in the community.

A second limitation is that, although IeDEA spans three countries, the patients from each country may not be representative of the country as a whole in all aspects. We believe, however, that sites are in general prototypical ART scale-up clinics staffed and stocked by national ministries of health and implementing partners of the Global Fund and PEPFAR and should be fairly representative of clinics in these countries. Third, the data we analyzed were collected in the course of routine clinical care, and the accuracy of certain measurements, such as WHO staging, may be limited by few diagnostic options. This may explain why a later calendar year was associated with a slightly high proportion of patients classified as WHO Stage 4 in Uganda even though the CD4 count levels rose during that interval.

## Conclusions

In summary, this study found encouraging trends over time in east Africa where scale up of ART services has expanded rapidly; however, gaps in effectiveness continue to exist. Patients are starting less toxic ART regimens, at clinics which are closer to their residences, free of charge and at higher CD4 count levels. The improving characteristics at ART initiation can be expected to have substantial effects on morbidity and mortality.

Areas that require further study and action include evaluation of why men present with advanced disease despite the fact that they control more resources in the community. Although CD4 cell count levels at ART initiation have risen, the average is still under 200 cells/mm^3^, and continued monitoring is needed to document further rises now that national guidelines have moved to a threshold of 350 cells/mm^3^. D4T use remains too high and implementers must move towards systematic reduction of D4T use whenever possible.

## Competing interests

The authors declare that they have no competing interests.

## Authors' contributions

EHG contributed to conception and design of the study, acquisition of data, data analysis, interpretation of data, drafting the manuscript, and critical revisions of the manuscript. PWH contributed to conception and design of the study, data analysis, interpretation of data, drafting the manuscript, and critical revisions of the manuscript. LOD contributed to conception and design of the study, acquisition of data, interpretation of data, and critical revisions of the manuscript GRS contributed to conception and design of the study, acquisition of data, interpretation of data, drafting the manuscript, and critical revisions of the manuscript. SK contributed to acquisition of data, and critical revisions of the manuscript. PO contributed to acquisition of data, interpretation of data, and critical revisions of the manuscript. DRB contributed to acquisition of data, interpretation of data, and critical revisions of the manuscript. MBB contributed to acquisition of data, interpretation of data, and critical revisions of the manuscript. CRC contributed to acquisition of data, interpretation of data, and critical revisions of the manuscript. JAO contributed to acquisition of data, interpretation of data, drafting the manuscript, and critical revisions of the manuscript. DW contributed to acquisition of data, data analysis, interpretation of data, drafting the manuscript, and critical revisions of the manuscript. BE contributed to acquisition of data, interpretation of data, and critical revisions of the manuscript. DN contributed to conception and design of the study, acquisition of data, interpretation of data, drafting the manuscript, and critical revisions of the manuscript. PJE contributed to acquisition of data, data analysis, interpretation of data, drafting the manuscript, and critical revisions of the manuscript. PB contributed to acquisition of data, interpretation of data, and critical revisions of the manuscript. BSM contributed to conception and design of the study, acquisition of data, interpretation of data, drafting the manuscript, and critical revisions of the manuscript JNM contributed to conception and design of the study, acquisition of data, data analysis, interpretation of data, drafting the manuscript, and critical revisions of the manuscript. CTY contributed to conception and design of the study, acquisition of data, interpretation of data, drafting the manuscript, and critical revisions of the manuscript. KWK contributed to conception and design of the study, acquisition of data, data analysis, interpretation of data, drafting the manuscript and critical revisions of the manuscript. All authors have read and approved the final manuscript.

## References

[B1] Office of the United States Global AIDS Coordinator. The Power of Partnerships: The President's Emergency Plan for AIDS Relief Fifth Annual Report to Congress 2009 2009 http://www.pepfar.gov/documents/organization/81019.pdf Accessed July 21, 2010

[B2] AIDS epidemic update December 2009 http://data.unaids.org/pub/Report/2009/JC1700_Epi_Update_2009_en.pdf

[B3] AIDS epidemic update December 2010 http://www.who.int/hiv/pub/2010progressreport/report/en/index.html

[B4] Thirumurthy H Goldstein M Graff Zivin J The economic impact of AIDS treatment: labor supply in western Kenya Journal of Human Resources 2008 14 511 552 22180664 PMC3237059

[B5] Makombe SD Jahn A Tweya H Chuka S Yu JK Hochgesang M Aberle-Grasse J Thambo L Schouten EJ Kamoto K Harries AD A national survey of teachers on antiretroviral therapy in Malawi: access, retention in therapy and survival PLoS One 2007 14 e620 10.1371/journal.pone.0000620 17637836 PMC1905945

[B6] Mermin J Were W Ekwaru JP Moore D Downing R Behumbiize P Lule JR Coutinho A Tappero J Bunnell R Mortality in HIV-infected Ugandan adults receiving antiretroviral treatment and survival of their HIV-uninfected children: a prospective cohort study Lancet 2008 14 752 759 10.1016/S0140-6736(08)60345-1 18313504

[B7] Graff Zivin J Goldstein M Thirumurthy H AIDS treatment and intrahousehold resource allocation: children's nutrition and schooling Journal of Public Economics 2009 14 1008 1015 10.1016/j.jpubeco.2009.03.003 PMC3238680 22180689

[B8] Stevens W Kaye S Corrah T Antiretroviral therapy in Africa Bmj 2004 14 280 282 10.1136/bmj.328.7434.280 14751902 PMC324462

[B9] Lawn SD Harries AD Anglaret X Myer L Wood R Early mortality among adults accessing antiretroviral treatment programmes in sub-Saharan Africa AIDS 2008 14 1897 1908 10.1097/QAD.0b013e32830007cd 18784453 PMC3816249

[B10] Lawn SD Bekker LG Myer L Orrell C Wood R Cryptococcocal immune reconstitution disease: a major cause of early mortality in a South African antiretroviral programme Aids 2005 14 2050 2052 10.1097/01.aids.0000191232.16111.f9 16260920

[B11] Otieno V Marima R Odhiambo J Penner J Ooko H Agot K Bukusi E Cohen C Improving enrollment and retention of youth into HIV services: lessons learned from Kisumu, Kenya17th International AIDS Conference; August 3 - 8, 2008; Mexico City 2008

[B12] Amuron B Namara G Birungi J Nabiryo C Levin J Grosskurth H Coutinho A Jaffar S Mortality and loss-to-follow-up during the pre-treatment period in an antiretroviral therapy programme under normal health service conditions in Uganda BMC Public Health 2009 14 290 10.1186/1471-2458-9-290 19671185 PMC2734853

[B13] Tuller DM Bangsberg DR Senkungu J Ware NC Emenyonu N Weiser SD Transportation Costs Impede Sustained Adherence and Access to HAART in a Clinic Population in Southwestern Uganda: A Qualitative Study AIDS Behav 2009 10.1007/s10461-009-9533-2 PMC2888948 19283464

[B14] World Health Organization HIV/AIDS Programme. Antiretroviral Therapy for Adults and Adolescents: Recommendations for a Public Health Approach 2010 Revision 2010 http://www.who.int/hiv/pub/guidelines/en/Accessed Sept. 20, 2010 23741771

[B15] Gilks CF Crowley S Ekpini R Gove S Perriens J Souteyrand Y Sutherland D Vitoria M Guerma T De Cock K The WHO public-health approach to antiretroviral treatment against HIV in resource-limited settings Lancet 2006 14 505 510 10.1016/S0140-6736(06)69158-7 16890837

[B16] Diez-Roux AV Bringing context back into epidemiology: variables and fallacies in multilevel analysis Am J Public Health 1998 14 216 222 10.2105/AJPH.88.2.216 9491010 PMC1508189

[B17] Diez-Roux AV Multilevel analysis in public health research Annu Rev Public Health 2000 14 171 192 10.1146/annurev.publhealth.21.1.171 10884951

[B18] Zou G A modified poisson regression approach to prospective studies with binary data Am J Epidemiol 2004 14 702 706 10.1093/aje/kwh090 15033648

[B19] Oyugi JH Byakika-Tusiime J Ragland K Laeyendecker O Mugerwa R Kityo C Mugyenyi P Quinn TC Bangsberg DR Treatment interruptions predict resistance in HIV-positive individuals purchasing fixed-dose combination antiretroviral therapy in Kampala, Uganda Aids 2007 14 965 971 10.1097/QAD.0b013e32802e6bfa 17457090

[B20] Kigozi IM Dobkin LM Martin JN Geng EH Muyindike W Emenyonu NI Bangsberg DR Hahn JA Late-disease stage at presentation to an HIV clinic in the era of free antiretroviral therapy in Sub-Saharan Africa J Acquir Immune Defic Syndr 2009 14 280 289 10.1097/QAI.0b013e3181ab6eab 19521248 PMC2815238

[B21] Cornell M Grimsruda A Fairall F Fox MP van Cutsem G Giddy J Wood R Prozesky H Mohapi L Graber C Temporal changes in programme outcomes among adult patients initiating antiretroviral therapy across South Africa, 2002-2007 AIDS 2010 14 2263 2270 10.1097/QAD.0b013e32833d45c5 20683318 PMC2948209

[B22] Egger M May M Chene G Phillips AN Ledergerber B Dabis F Costagliola D D'Arminio Monforte A de Wolf F Reiss P Prognosis of HIV-1-infected patients starting highly active antiretroviral therapy: a collaborative analysis of prospective studies Lancet 2002 14 119 129 10.1016/S0140-6736(02)09411-4 12126821

[B23] Westreich DJ Sanne I Maskew M Malope-Kgokong B Conradie F Majuba P Funk MJ Kaufman JS Van Rie A Macphail P Tuberculosis treatment and risk of stavudine substitution in first-line antiretroviral therapy Clin Infect Dis 2009 14 1617 1623 10.1086/598977 19385733 PMC2787193

[B24] Cohen K van Cutsem G Boulle A McIlleron H Goemaere E Smith PJ Maartens G Effect of rifampicin-based antitubercular therapy on nevirapine plasma concentrations in South African adults with HIV-associated tuberculosis J Antimicrob Chemother 2008 14 389 393 18096560 10.1093/jac/dkm484

[B25] Manosuthi W Sungkanuparph S Tantanathip P Lueangniyomkul A Mankatitham W Prasithsirskul W Burapatarawong S Thongyen S Likanonsakul S Thawornwa U A randomized trial comparing plasma drug concentrations and efficacies between 2 nonnucleoside reverse-transcriptase inhibitor-based regimens in HIV-infected patients receiving rifampicin: the N2R Study Clin Infect Dis 2009 14 1752 1759 10.1086/599114 19438397

